# An Amide-Carboxylic Acid Compound as Gel Structure Breaker to Improve the Rheology of Oil-Based Drilling Fluids

**DOI:** 10.3390/gels11020097

**Published:** 2025-01-30

**Authors:** Yu Zhang, Kaihe Lv, Xianbin Huang, Zhe Li, Yang Zhang, Zhenhang Yuan

**Affiliations:** 1 Key Laboratory of Unconventional Oil & Gas Development, China University of Petroleum (East China), Qingdao 266580, China; z22020051@s.upc.edu.cn (Y.Z.); 17864203250@163.com (Z.L.); z23020057@s.upc.edu.cn (Y.Z.); z23020045@s.upc.edu.cn (Z.Y.); 2 School of Petroleum Engineering, China University of Petroleum (East China), Qingdao 266580, China

**Keywords:** rheology modifier, gel structure breaker, amide-carboxylic acid compound, high-density oil-based drilling fluid

## Abstract

High-density oil-based drilling fluids (OBDFs) are widely used in drilling operations, but during their application, the viscosity of the fluid typically increases due to the enhancement of the solid-phase gel network structure. This can lead to issues such as impaired fluid circulation, increased blowout risks, and accelerated drill bit wear. In this study, a compound (OCD), synthesized from tall oil fatty acids, diethylene triamine, and maleic anhydride, was developed to disrupt the strong gel structure in high-density OBDFs, thereby reducing the viscosity of the OBDFs. Rheological properties, including viscosity, yield point, and gel strength, were tested to evaluate the viscosity-reducing effect of OCD on both laboratory-prepared and field high-density OBDFs. Additionally, the effects of OCD on electrical stability (ES), high-temperature high-pressure (HTHP) filtration loss, and solid-phase settling stability were also tested. Finally, the mechanism of OCD was analyzed through contact angle tests, particle size analysis, and microstructural observations. The experimental results demonstrated that OCD could effectively reduce the viscosity of various high-density OBDFs. Adding 2 wt% of OCD reduced the apparent viscosity of laboratory-prepared OBDFs by 20.4%, and reduced the apparent viscosity of field OBDFs with a density of 1.7 g/cm^3^ by 29.2%. Furthermore, OCD showed good compatibility with OBDFs, having negligible effects on HTHP filtration loss and ES, and maintained good viscosity-reducing performance even at 180 °C. Mechanistic studies revealed that OCD enhanced the hydrophobicity of the solid phase, reduced the particle size of solids, and prevented the formation of excessive network structures in the oil. Therefore, this study provides significant practical value for controlling the rheological performance of the gel system in OBDFs.

## 1. Introduction

With the ongoing advancements in oil exploration and production technologies, especially the increasing demand for drilling in deep wells, high-temperature high-pressure wells, and complex geological conditions, the use of high-density OBDFs has become more widespread [1,2,3,4]. High-density OBDFs possess excellent properties, such as high-temperature resistance, lubrication, reservoir protection, and blowout control, playing a crucial role in deep and ultra-deep well drilling operations [5,6,7,8,9,10,11,12]. However, since high-density OBDFs typically achieve the required density by adding premium barite particles, they inherently have a high solid-phase content. Additionally, the continuous invasion of deleterious solid phases during the circulation drilling process causes the viscosity of the system to increase. This is because the harmful solid phases and barite in OBDFs are usually irregularly shaped particles, which can easily interact with other particles and droplets, forming a spatial network structure with a certain gel strength [13]. Excessively high viscosity not only increases the flow resistance of the fluid during drilling, consuming more mechanical energy, but may also reduce the carrying capacity of the drilling fluid, accelerate drill bit wear, and lead to excessive pumping pressure, which severely affect drilling efficiency and safety. Therefore, effectively controlling and optimizing the rheological properties of high-density OBDFs to improve their reusability has become one of the key challenges in advancing drilling technology.

Currently, the main viscosity-reducing methods used in drilling operations include mechanical removal of solids or diluting the thickened old mud with fresh mud [14]. However, diluting thickened mud with fresh mud requires a large amount of new mud, which increases the volume of drilling fluid and raises transportation and storage costs. Furthermore, this viscosity reduction method also increases the amount of oil-based mud that needs to be treated later, posing significant environmental challenges [15,16,17]. Additionally, when the OBDF system contains a large amount of submicron or nanoscale solid particles, traditional solid control equipment often cannot effectively remove these particles. In such cases, using viscosity reducers to improve the rheological properties of OBDFs, enabling their recycling, and reducing drilling costs, becomes an ideal solution.

In past studies, the application of viscosity reducers has mainly focused on water-based drilling fluids, while research on viscosity reducers for high-density OBDFs is still insufficient [18,19,20,21]. MING Xiansen et al. [22] developed a viscosity reducer, CQ-OTA, with amine and amide groups in its molecular structure, using lauramide, stearamide, and erucamide as raw materials. CQ-OTA has been applied in the Wei-202HX platform, where it reduced the plastic viscosity of the OBDF from 53.0 mPa·s to 40.0 mPa·s, and the gel strength at 10 min from 23 Pa to 14.5 Pa, ensuring successful drilling and improving the recycling efficiency of high-density OBDFs. Kania D. et al. [23]. studied the performance of polyol esters as diluents and lubricating enhancers in inverse emulsion drilling fluids (SBM). After 135 °C thermal rolling, polyol esters significantly reduced the rheological properties of the drilling fluid, and fitting the data to the Herschel–Bulkley model showed a decrease in yield stress. Although some products have achieved effective results in improving the rheology of OBDFs, the compatibility between viscosity reducers and OBDFs still needs further enhancement.

This study developed a carboxylic acid amide-based compound (OCD) as a viscosity reducer for high-density OBDFs. The OCD is synthesized through a two-step reaction using tall oil fatty acids, diethylene triamine, and maleic anhydride as reactants. OCD has a surfactant-like molecular structure composed of both hydrophobic and hydrophilic groups. Through this amphiphilic structure, OCD alters the interactions between solid particles in the drilling fluid, disrupting their aggregation structure or reducing the adhesion between particles, thereby achieving a viscosity-reducing effect. The rheological properties of high-density OBDFs, including apparent viscosity (AV), plastic viscosity (PV), yield point (YP), gel strength at 10 s (Gel_10s_), and gel strength at 10 min (Gel_10min_) and the rheological plastic ratio (YP/PV), were tested before and after the addition of OCD, demonstrating its excellent improvement in rheological properties. Additionally, high-temperature, high-pressure (HTHP) filtration loss and electrical stability (ES) measurements were conducted, showing that OCD has minimal impact on filtration loss and ES, with good compatibility with the drilling fluid system. Finally, the viscosity-reducing mechanism of OCD was analyzed through solid-phase water contact angle experiments, particle size distribution tests, and microstructural observations. Through systematic experimental studies and performance evaluations, this paper provides technical support for the research of viscosity reducers for high-density OBDFs and promotes the development of efficient drilling technologies.

## 2. Results and Discussion

### 2.1. FTIR

**Figure 1:**
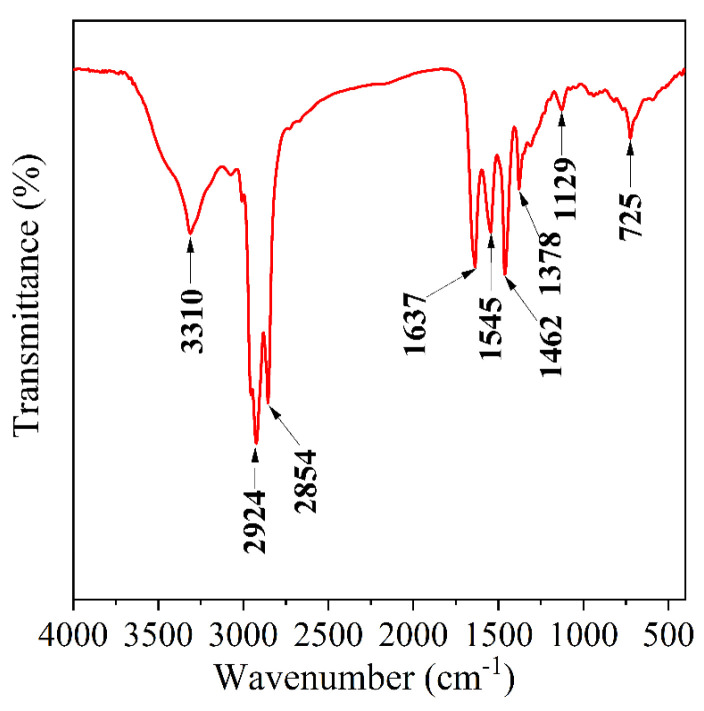
Infrared spectral analysis of OCD.

FTIR can be used to analyze the functional groups contained in compounds to determine whether the synthesized material is the target product [24]. The spectrum of OCD is shown in Figure 1. The peak at 3310 cm^−1^ is attributed to the stretching vibrations of N–H and O–H. The absorption peaks at 2924 cm^−1^ and 2854 cm^−1^ correspond to the stretching vibrations of C–H. The absorption peak at 1637 cm^−1^ is associated with the stretching vibration of C=O (amide I band) and the C=O stretching vibration of the carboxylate group. The absorption peak at 1527 cm^−1^ results from the bending vibration of N–H and the stretching vibration of C=C. The absorption peak at 1462 cm^−1^ is linked to the bending vibration of –CH_2_–, and the absorption peak at 1378 cm^−1^ corresponds to the bending vibration of –CH_2_–. Additionally, the bending vibrational absorption peak of –CH_2_– appears at 1462 cm^−1^, while the absorption peak at 1378 cm^−1^ is associated with the C–N stretching vibration (amide III band). The in-plane bending vibrational absorption peak of C–H appears at 1129 cm^−1^, and the out-of-plane bending vibrational absorption peak of C–H is observed at 725 cm^−1^. The infrared spectrum indicates that the synthesized OCD molecular structure contains the designed functional groups, confirming that the synthesized product achieved the intended molecular structure design.

### 2.2. Performance Evaluation of OCD

#### 2.2.1. Effect of OCD on the Rheological Properties of Laboratory-Prepared OBDFs

**Figure 2:**
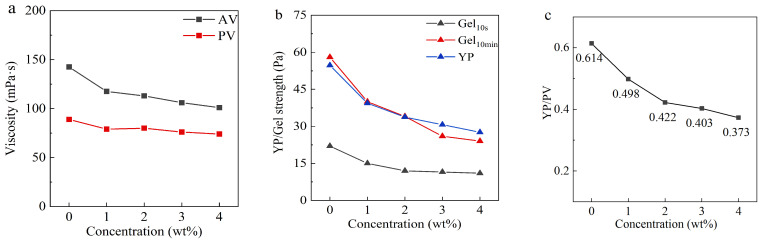
Relationship between OCD concentration and AV (**a**), PV (**a**), YP (**b**), Gel_10s_ (**b**), Gel10_min_ (**b**), and YP/PV (**c**) of laboratory-prepared OBDFs.

The variation curves of viscosity, yield point (YP), gel strength, and rheological plastic ratio (YP/PV) of drilling fluid under different OCD concentrations are shown in Figure 2. Due to the high density and solid-phase content of this drilling fluid system, the interactions between solid particles are significant, resulting in high viscosity, YP, and gel strength. However, with the gradual increase in OCD concentration, the rheological properties and internal structure of the drilling fluid undergo significant changes. Specifically, key indicators such as apparent viscosity (AV), plastic viscosity (PV), YP, 10 s gel strength (Gel_10s_), 10 min gel strength (Gel_10min_), and YP/PV all exhibit a gradual decreasing trend.

It is worth noting that when the concentration of OCD exceeds 2 wt%, the rate of reduction in the viscosity and shear force of the drilling fluid begins to slow down. Moreover, from an economic perspective, we recommend an OCD addition amount of 2 wt%. When the OCD concentration increases to 2 wt%, the AV of the drilling fluid decreases sharply from 142 mPa·s to 113 mPa·s, a reduction of 20.4%. This significant change clearly indicates that at 2 wt% concentration, OCD can effectively reduce the overall viscosity of the drilling fluid, greatly improving its flowability. Meanwhile, the PV decreases from 89 mPa·s to 80 mPa·s, with a smaller reduction of 4.5%, yet still showing that OCD has an effect in reducing the friction between solid particles in the drilling fluid, thus improving the pumpability and flow properties of the drilling fluid.

Furthermore, as the OCD concentration increases to 2 wt%, the gel strength of the drilling fluid also shows a significant decrease. Specifically, Gel_10s_ decreases from 22 Pa to 12 Pa, a reduction of 45.5%. Gel_10min_ decreases from 58 Pa to 34 Pa, a decrease of 41.4%. These changes demonstrate that OCD can effectively disrupt the gel structure of the drilling fluid, significantly enhancing its flowability and reducing the risk of settling and blockage when the fluid is at rest. The YP also decreases from 54.677 Pa to 33.726 Pa, a decrease of 38.3%, further confirming the effectiveness of OCD in reducing the gel strength of the drilling fluid. As the OCD concentration increases to 2 wt%, YP/PV decreases from 0.614 to 0.422. This change occurs because OCD mainly adjusts the rheological plastic ratio by reducing the YP, thereby improving the overall rheological properties of the drilling fluid. The decrease in the YP/PV ratio enables the drilling fluid to start flowing more easily under lower pressure. In actual drilling operations, this means that when the drilling fluid flows through the pipe string and annulus, the starting pump pressure required is lower, which can effectively reduce the energy consumption of the pump. Although the YP/PV ratio has decreased, it can still be kept above 0.3. Therefore, OCD significantly improves the rheological properties of the OBDF while ensuring that the drilling fluid has certain cuttings carrying performance.

In summary, with the continuous increase in OCD concentration, the rheological properties and internal structure of the drilling fluid have been significantly improved. As a gel breaker, OCD successfully reduced the drilling fluid’s viscosity, YP, and gel strength, significantly enhancing its flowability and pumpability.

#### 2.2.2. Effect of OCD on the Rheological Properties of Field OBDFs with Different Densities

**Figure 3:**
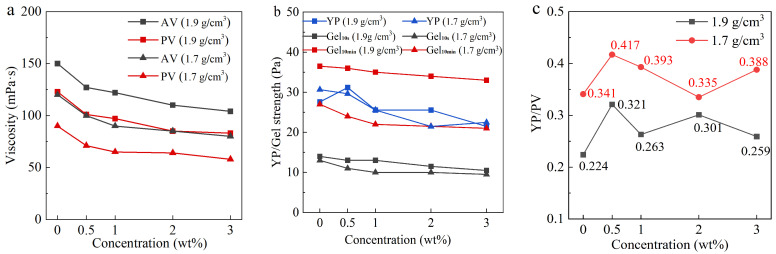
Relationship between OCD concentration and AV (**a**), PV (**a**), YP (**b**), Gel_10s_ (**b**), Gel_10min_ (**b**), and YP/PV (**c**) of field OBDFs.

The effects of OCD on the AV, PV, YP, Gel_10s_, and Gel_10min_ of OBDFs are shown in Figure 3. As the OCD concentration increases, the viscosity of OBDFs decreases significantly. Similarly, considering that when the concentration of OCD exceeds 2 wt%, the reduction rate of the viscosity and shear force of the drilling fluid will slow down. And, in order to improve the economic benefits of on-site applications, we recommend the optimal concentration of OCD to be 2 wt%. When the OCD concentration increases to 2 wt%, the AV of the drilling fluid with a density of 1.9 g/cm^3^ decreases from 150 mPa·s to 110 mPa·s, a reduction of 26.7%, the PV decreases from 123 mPa·s to 85 mPa·s, a reduction of 30.9%. For the drilling fluid with a density of 1.7 g/cm^3^, the AV decreases from 120 mPa·s to 85 mPa·s, a reduction of 29.2%, the PV decreases from 90 mPa·s to 64 mPa·s, a reduction of 28.9%. The significant decrease in PV indicates that OCD effectively reduces the internal friction of the drilling fluid, thereby reducing wear on equipment and energy consumption during drilling.

Additionally, as shown in Figure 3b, with the increase in OCD concentration, the Gel_10s_, Gel_10min_, and YP of both OBDFs also show slight decreases. When the OCD concentration increases to 2 wt%, the Gel_10s_ of the drilling fluid with a density of 1.9 g/cm^3^ decreases from 14 Pa to 11.5 Pa, a decrease of 17.9%; the Gel_10min_ decreases from 36.5 Pa to 34 Pa, a decrease of 6.8%; and the YP decreases from 27.594 Pa to 25.55 Pa, a decrease of 7.4%. For the drilling fluid with a density of 1.7 g/cm^3^, the Gel_10s_ decreases from 13 Pa to 10 Pa, a decrease of 23.1%; the Gel_10min_ decreases from 27 Pa to 21.5 Pa, a decrease of 20.4%; and the YP decreases from 30.66 Pa to 21.462 Pa, a decrease of 30%. These experimental results confirm that OCD effectively reduces the gel strength of both types of OBDFs, further enhancing their flowability.

Figure 3c shows that OCD has a minimal effect on the rheological plastic ratio of the two OBDFs, and it essentially does not alter the rock carrying capacity of the drilling fluid.

Although the desulfurization effects of OCD on the two OBDFs differ slightly, OCD has a significant impact on high-density, high-viscosity OBDFs. The significant reduction in key parameters such as AV, PV, and gel strength indicates that OCD can effectively improve the flow properties and pumpability of high-density drilling fluids.

#### 2.2.3. Effect of OCD on the HTHP Filtration and Electrical Stability of OBDFs

**Figure 4:**
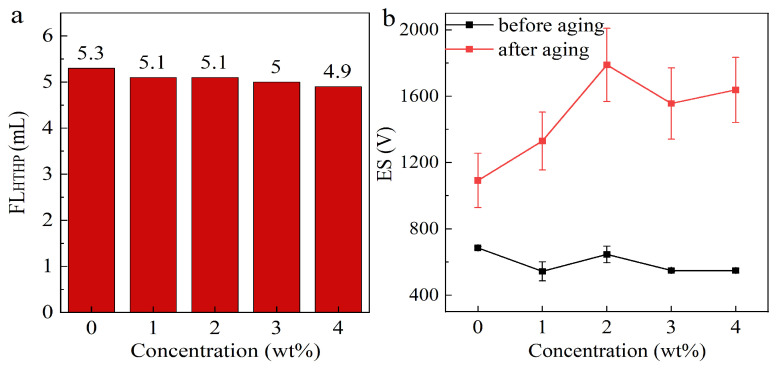
Relationship among OCD concentration, the HTHP filtration loss (**a**), and ES (**b**) of OBDF. The aging temperature is 135 °C.

**Figure 5:**
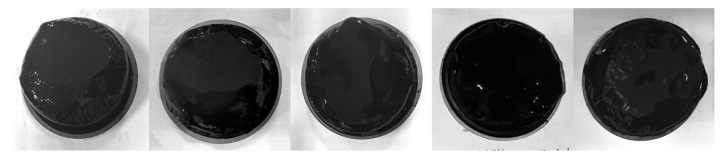
The mud cake images obtained from HTHP filtration tests of OBDFs with varying OCD contents. From **left** to **right**, the OCD content gradually increases.

The HTHP filtration loss of OBDF at different OCD concentrations is shown in Figure 4a. As the OCD concentration increases, the HTHP filtration loss of OBDF gradually decreases. This may be because OCD reduces the aggregation and settling of particles, which helps form a tighter and more uniform filter cake, as shown in Figure 5. A uniform and dense filter cake can effectively reduce the liquid penetration through the cake. When the OCD concentration increases from 0 to 2 wt%, the HTHP filtration loss of OBDF decreases from 5.2 mL to 4.9 mL, indicating that OCD has no negative effect on the HTHP filtration loss of OBDF. This helps maintain the stability of the wellbore and reduces damage to the formation.

In addition, emulsion stability (ES) is also one of the most important properties of OBDF. The effect of OCD on the electrical stability of the laboratory-prepared OBDF is shown in Figure 4b. Before aging, increasing the concentration of OCD in the OBDF slightly reduced the electrical stability, but it remained above 400 V. The effect of OCD on the electrical stability of the aged OBDF was more pronounced. As the OCD concentration increased, the electrical stability of the OBDF shows an overall upward trend. This could be attributed to the fact that OCD migrates to the oil–water interface and reduces the interfacial tension. This alteration in interfacial tension stabilizes the emulsion droplets, making them more resistant to disruption, thereby requiring a higher voltage to break the more stable emulsion structure during the emulsion-breaking process.

#### 2.2.4. Evaluation of Temperature Resistance of OCD

The laboratory-prepared OBDFs with different OCD OBDFs are used at high temperatures, so the thermal stability of additives is crucial to the performance of the drilling fluid [25,26]. To comprehensively evaluate the thermal resistance of OCD, the effects of OCD on the viscosity, electrical stability, and HTHP filtration loss of the drilling fluid at different aging temperatures were tested, and results are shown in Figure 5.

**Figure 6:**
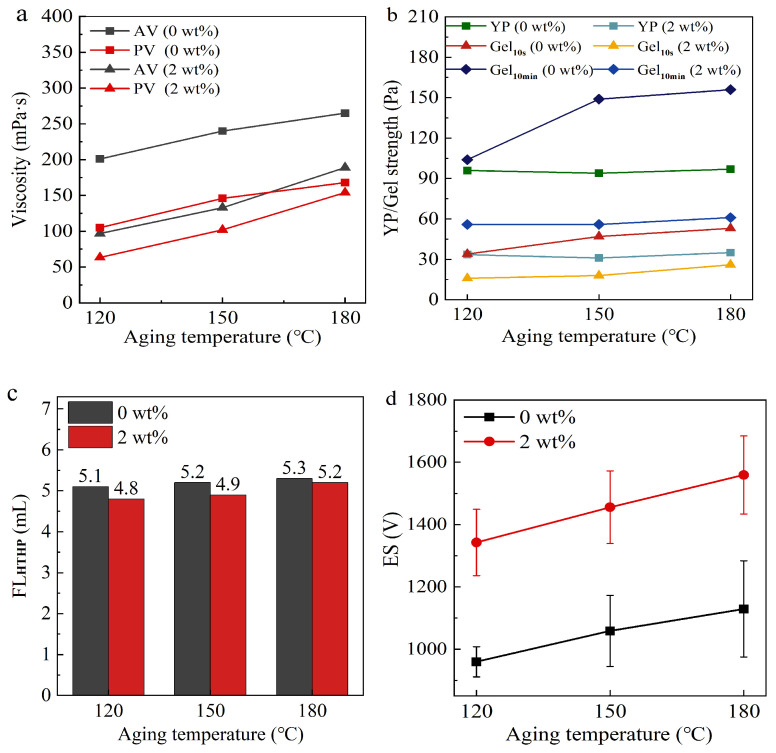
The effects of OCD on the rheological properties (**a**,**b**), HTHP filtration (**c**), and electrical stability (**d**) of OBDF at different aging temperatures.

As can be seen from Figure 6a,b, with the increase in aging temperature, the viscosity of the OBDF increased sharply. This can be ascribed to the enhanced interactions between solid-phase particles at high temperatures [27]. For example, bitumen in drilling fluids often contains large molecular structures. As the temperature rises, bitumen softens and undergoes structural changes, potentially forming polymer chains or network structures. These chain-like structures may adsorb onto the surface of solid-phase particles, increasing the attraction between the particles and making them more prone to aggregation or coagulation. This aggregation effect could lead to an increase in the viscosity of the drilling fluid.

As shown in Figure 6a, the AV of the OBDF increased to 240 mPa·s, and the PV increased to 146 mPa·s after aging at 150 °C. However, when 2 wt% OCD was added, a significant change in viscosity was observed. After adding 2 wt% OCD, the AV decreased to 133 mPa·s, a reduction of 44.6%, and the PV decreased to 102 mPa·s, a reduction of 30.1%. Similarly, under high-temperature conditions, OCD effectively reduced the YP and gel strength of the drilling fluid. After 16 h of aging at 150 °C, the YP of the OBDF was 94 Pa, with the Gel_10s_ at 47 Pa and Gel_10min_ at 149 Pa. For the drilling fluid with 2 wt% OCD, the YP decreased to 31 Pa, the Gel_10s_ dropped to 18 Pa, and the Gel_10min_ decreased to 56 Pa. These results demonstrate that even under high-temperature conditions, OCD can effectively maintain its viscosity-reducing effect.

From Figure 6c, it can be seen that the HTHP filtration loss of the drilling fluid shows a gradual upward trend with the increase in aging temperature. However, compared to the OBDF without OCD, the fluid with OCD exhibited lower HTHP filtration loss. Under aging conditions at 180 °C, the HTHP filtration loss of the base OBDF was 5.4 mL, while the fluid containing 2 wt% OCD reduced to 5.2 mL. This indicates that the addition of the viscosity-reducing agent does not adversely affect the filtration performance of the base OBDF and, to some extent, improves its performance.

Meanwhile, the electrical stability of the OBDF increases gradually with the aging temperature. As shown in Figure 6d, after aging at 150 °C, the electrical stability of the base OBDF was 1058 V, whereas that of the fluid containing 2 wt% OCD significantly increased to 1456 V. This comparison demonstrates that OCD exhibits excellent thermal stability under high-temperature conditions, effectively maintaining the electrical stability of the drilling fluid and ensuring its reliable performance during the drilling process.

#### 2.2.5. OCD’s Effect on the Solid-Phase Settling Stability

**Figure 7:**
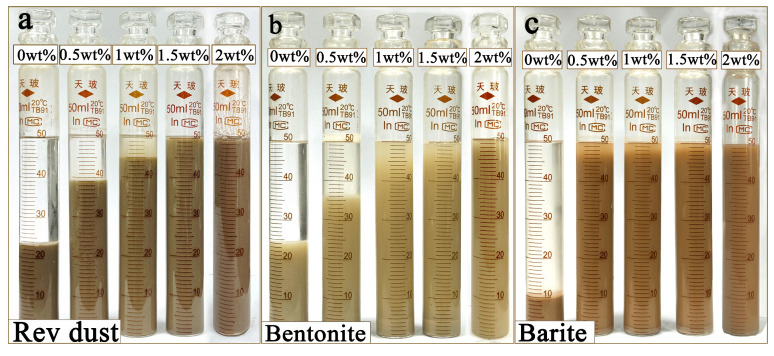
Rev dust (**a**), bentonite (**b**), and barite (**c**) show changes in settling stability in white oil suspensions with different OCD concentrarions. The Chinese text in the figure indicates the manufacturer of the graduated cylinder and has no special significance.

Due to the presence of a large amount of weighting agents (such as barite) in high-density OBDFs, these solid particles often have a significant density difference in the oil phase, which makes them prone to settling [28,29]. The settling of solid particles not only affects the rheological properties of the drilling fluid but may also reduce the fluid’s suspension capability, thereby impacting the efficiency and safety of the drilling operation. This section evaluates the effect of OCD on the settling stability of solid phases in oil, as shown in Figure 7. From Figure 7, it can be seen that the addition of OCD significantly improved the settling stability of Rev dust, bentonite, and barite in oil, which could be attributed to the amphipathic structure of OCD enhancing the oil affinity of solid phases, thereby promoting better dispersion in the oil phase.

### 2.3. Mechanism Analysis

In high-density OBDFs, solid particles (particularly hydrophilic detrimental solids such as clay and weighting materials like barite) significantly affect the rheological properties of the drilling fluid. Due to the strong hydrophilicity of these solid phases, they are difficult to disperse in the oil phase, often forming flocculation or aggregation that leads to the formation of a three-dimensional gel network structure, increasing the viscosity of the fluid. Furthermore, during the circulation of OBDFs, the hydrophilic solid phases in the system may adsorb a water film on their surfaces, further increasing the friction between the solids and the oil, resulting in higher viscosity. To address this issue, OCD interacts with the hydrophilic groups on the surface of the solid phases through its own hydrophilic groups, which allows OCD to adsorb onto the solid-phase particles, while the hydrophobic long carbon chains in its molecular structure orient towards the oil phase, thereby enhancing the hydrophobicity of the solid phase. This effectively reduces intermolecular forces between particles, breaks the gel network structure, and weakens particle aggregation.

**Figure 8:**
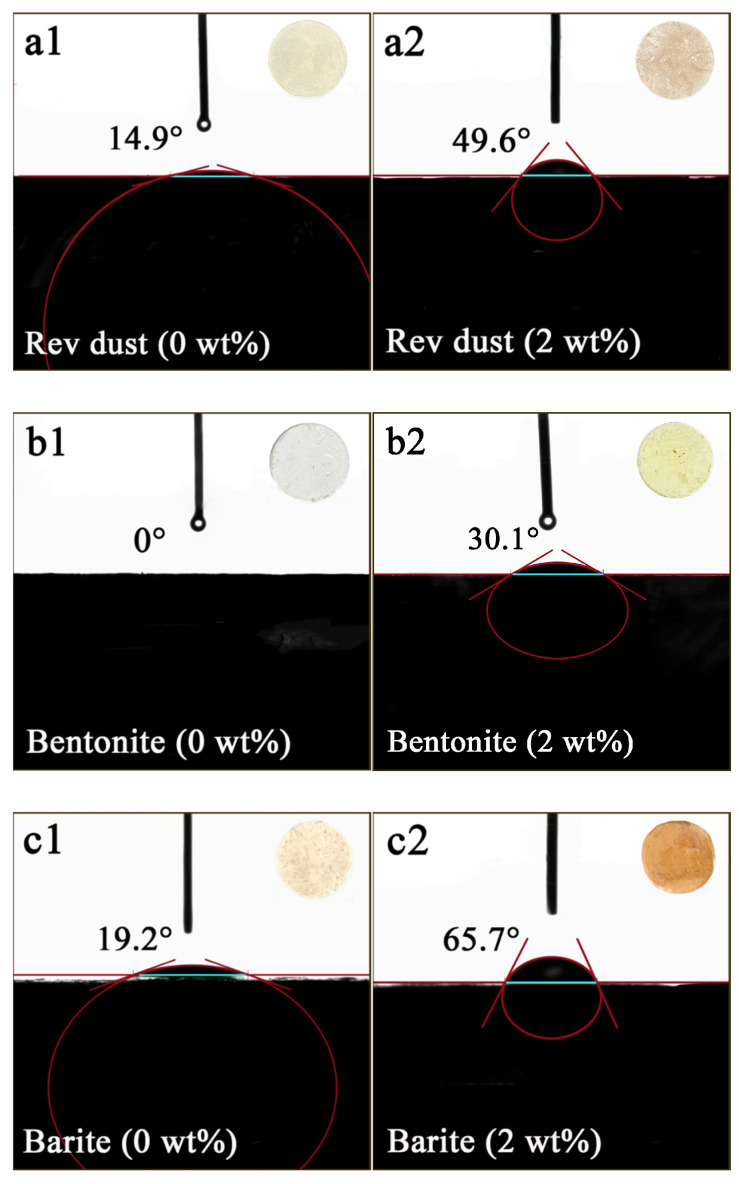
The effect of OCD on the water contact angles of Rev dust (**a**), bentonite (**b**), and barite (**c**). (**a1**–**c1**) represent the samples without OCD, while (**a2**–**c2**) correspond to the samples with 2 wt% OCD. The upper right corner of the image shows the solid-phase tablet. The error in the angle is ±0.5°.

As shown in Figure 8, the contact angle of Rev dust with water was measured to be 14.9°. However, when the OCD concentration increased to 2 wt%, the contact angle of Rev dust with water changed to 49°. Bentonite, which has good hydrophilicity, has a contact angle close to 0° with water, but the contact angle increases to 30° when the OCD concentration increased to 2 wt%. Barite has a contact angle of 19.2° with water, which increases to 65.7° after the addition of OCD. These results indicate that OCD could adsorb onto the surface of solid phases and reduce their hydrophilicity.

**Figure 9:**
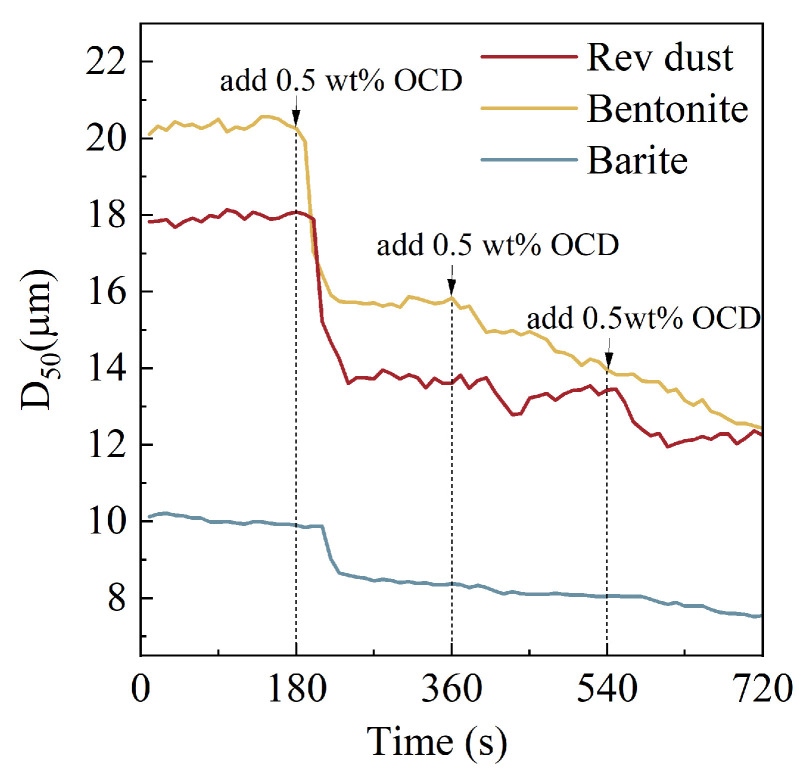
The effect of OCD on the particle size of Rev dust, bentonite, and barite in white oil.

As shown in Figure 9, with the increase in OCD concentration, the median particle size (D_50_) of Rev dust, barite, and bentonite gradually decreases. This indicates that OCD effectively weakens the interactions between solid particles, disrupting the gel network structure formed between the particles and promoting a more uniform dispersion of solid particles in the oil phase. After adding 0.5 wt% OCD, the D_50_ of Rev dust decreases from 18.08 μm to 13.76 μm, the D_50_ of bentonite reduces from 20.26 μm to 15.83 μm, and the D_50_ of barite decreases from 9.96 μm to 8.32 μm. Compared to barite particles, the reduction in particle size of bentonite and Rev dust is more pronounced under specific conditions, which is closely related to their unique physicochemical properties. Bentonite and Rev dust typically exhibit flaky structures with strong hydrophilicity and large specific surface areas, which make them prone to particle aggregation in the oil phase. Therefore, OCD interacts with the surfaces of these solid phases, enhancing their hydrophobicity and effectively reducing aggregation, resulting in a significant reduction in particle size. In contrast, barite, as an inert solid phase primarily composed of barium sulfate, has stable chemical properties, weak interactions between particles, and is less likely to react with other substances, making it less prone to agglomeration. As a result, the effect of OCD on the particle size of barite is relatively small.

**Figure 10:**
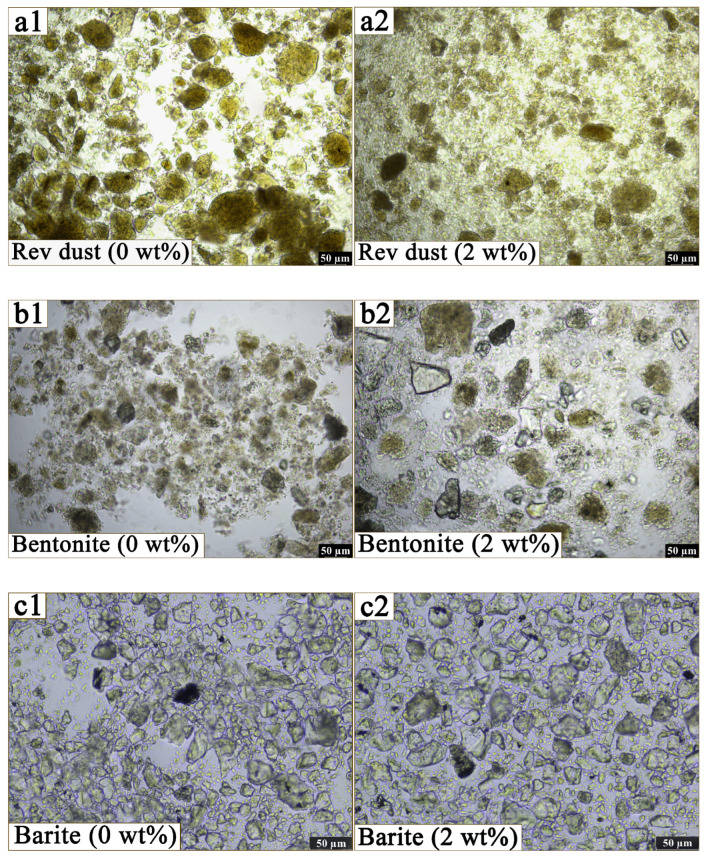
(**a1**–**c1**) show the microscopic morphology of Rev dust, bentonite, and barite in white oil before the addition of OCD, while subfigures (**a2**–**c2**) depict the morphological changes after the addition of 2 wt% OCD.

As shown in Figure 10, there are significant changes in the state of solid particles in the oil after the addition of OCD. Rev dust and bentonite are in an aggregated state in the oil phase, with a lamellar particle structure, as shown in Figure 10(a1,b1). The barite particles in the oil phase has poor size uniformity and distinct angular shapes, as seen in Figure 10(c1). However, after the addition of OCD, the surface of the Rev dust and bentonite particles becomes smoother, the gaps between the particles increases, and aggregation is significantly reduced, as shown in Figure 10(a2,b2). Barite particles are more evenly distributed in the oil phase after the addition of OCD, as shown in Figure 10(c2). The results indicate that OCD plays a positive role in enhancing the dispersion of clay particles.

**Figure 11:**
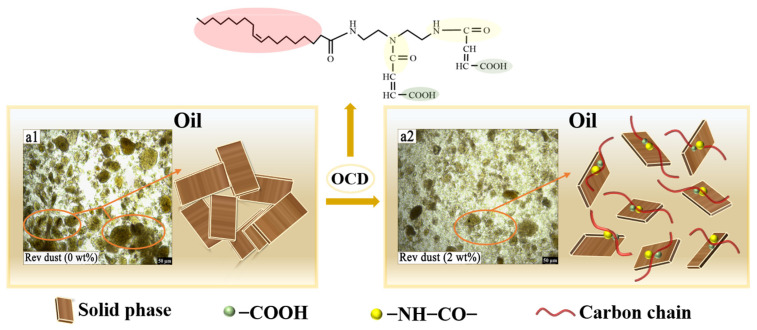
Analysis of the viscosity reduction mechanism of OCD.

In conclusion, the viscosity reduction mechanism of OCD mainly involves improving the hydrophobicity of solid phases, reducing intermolecular forces between particles, breaking the aggregation gel network between solids, and improving the dispersion of solids in the oil phase. This enhances the flowability and suspension stability of the drilling fluid. Additionally, the increased hydrophobicity of the hydrophilic solid phases reduces the thickness of the water film, thereby decreasing friction between the solid phases and the oil. The viscosity reduction mechanism of OCD is shown in Figure 11. These effects help reduce the viscosity of the drilling fluid, ensure its stability under high-temperature and high-pressure conditions, and improve its performance during the drilling process.

## 3. Conclusions

To address the issue of poor rheological properties in high-density OBDFs, this study synthesized a high-temperature-resistant viscosity reducer for OBDFs, named OCD. The evaluation results show that at a concentration of 2 wt%, OCD effectively reduces the viscosity, YP, and gel strength of three types of high-density OBDFs, with a viscosity reduction rate of over 20%. At 180 °C, OCD still demonstrates excellent performance. Additionally, OCD has minimal impact on the HTHP filtrate loss and ES of the OBDF, indicating good compatibility with the system. Mechanistic experiments show that OCD enhances the hydrophobicity of the solid phases by adsorbing onto the surface of hydrophilic solids, weakening the intermolecular forces between particles, disrupting the aggregation of these solids, and breaking the gel structure of the OBDF, thereby improving their dispersion and settling stability. At the same time, the enhanced hydrophobicity of the solid phases reduces the thickness of the water film adsorbed on the hydrophilic solid surface, decreasing the friction between the solid phases and the oil, thereby reducing the viscosity of the system. Based on these findings, the high-temperature-resistant, high-density OBDF viscosity reducer OCD shows great potential for application in the field of efficient drilling fluids.

## 4. Materials and Methods

### 4.1. Materials

Tall oil fatty acid (TOFA, 99 wt%), which is the source of the long-chain structure in OCD, was purchased from Guangzhou Fufei Chemical Technology Company. Diethylenetriamine (DETA, 99 wt%), which contains multiple amino groups (-NH_2_) and provides multiple reactive sites, was purchased from Shanghai Aladdin Biochemical Technology Co., Ltd., Shanghai, China. Maleic anhydride (MA, 99 wt%), which can introduce multiple polar groups into OCD, was purchased from Shanghai Aladdin Biochemical Technology Co., Ltd., Shanghai, China. The calcium oxide (CaO, 99 wt%), calcium chloride (CaCl_2_, 99 wt%), and anhydrous ethanol were purchased from Shanghai Aladdin Biochemical Technology Co., Ltd., Shanghai, China. The #5 white oil was purchased from Shenzhen ZRT Chemical Industry, Shenzhen, China. The bentonite, emulsifier, organoclay, asphalt, lignite and barite were provided by Petro China Great Wall Drilling Engineering Co., Ltd., Beijing, China. The on-site OBDFs and Rev dust (used to simulate deleterious solid phases) were provided by China Oilfield Services Co., Ltd., Tianjin, China.

### 4.2. Synthesis and Characterization of OCD

#### 4.2.1. OCD Synthesis

**Figure 12:**
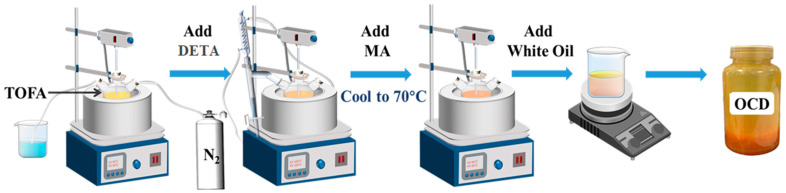
The synthesis process and reaction apparatus of OCD. The reaction temperatures at various stages are marked on the digital display of the oil bath.

**Scheme 1:**
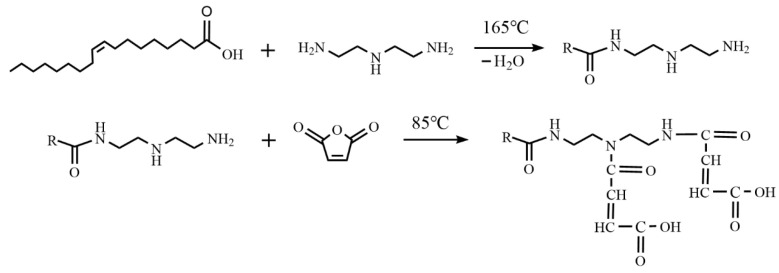
Synthesis of oil-based drilling fluid used rheological improver (OCD) using TOFA, DETA, and MA, and using a two-step method.

A mass of 63.4 g of TOFA was added to a three-necked flask and heated to 95 °C. Nitrogen was passed through the reaction setup for 30 min to ensure a pure reaction environment. Then, 33.4 g of DETA was added, and the temperature was increased to 165 °C. A water removal device was installed to continuously remove the water generated during the reaction. After 4 h of reaction, the temperature was lowered to 70 °C. When the intermediate product cooled to 70 °C, 7.4 g of MA was slowly added, and the temperature was adjusted to 85 °C. The reaction was continued for another 2 h. After the reaction was completed, the brown viscous product was poured out and diluted to 1.5 times its original volume with #5 white oil, and then stirred evenly. The resulting yellow liquid was the final product, OCD. The reaction equations are shown in Scheme 1, and the synthesis process of OCD is shown in Figure 12.

#### 4.2.2. OCD Characterization

OCD was characterized by the film-coating method using an infrared spectrometer (IRTRacer-100, Shimadzu Corporation, Kyoto, Japan). The liquid sample was uniformly coated on a transparent chlorinated polyethylene film to form a homogeneous liquid film on the sample. The scanning wavelength was 500~4000 cm^−1^ with a resolution of 4 cm^−1^.

### 4.3. Drilling Fluid Preparation and Performance Test

#### 4.3.1. Preparation of High-Density OBDFs

Table 1 focuses on the formulation of the oil-based drilling fluid, providing detailed information on the sequence, quantity, mixing time, and functions of each component. In these mud samples, white oil serves as the continuous phase, while water is the internal phase. Additionally, other functional additives are included to regulate the drilling fluid’s pH, fluid loss, filtrate volume, and other performance parameters. Laboratory-prepared OBDF was prepared according to Table 1.

#### 4.3.2. Rheological Performance Testing

Different concentrations of OCD were added to various oil-based drilling fluids and stirred at 10,000 rpm for 20 min to obtain the drilling fluid samples to be tested. The rheological parameters of the OBDF were measured using a six-speed rotational viscometer (ZNN-D6B, Tongchun Petroleum Instrument Co., Ltd., Qingdao, China) at 50 °C. The spring stiffness was increased to make the range of the six-speed rotational viscometer twice that of the standard range. Based on the measured data, the apparent viscosity (AV), plastic viscosity (PV), yield point (YP), 10 s gel strength (Gel_10s_) and 10 min gel strength (Gel_10min_) of the OBDF were calculated. The calculation formulas are provided in Equations (1)–(5), as follows:

(1) $$AV=0.5\Phi_{600}$$

(2) $$PV=\Phi_{600}-\Phi_{300}$$

(3) $$YP=0.511(\Phi_{300}-PV)$$

(4) $${Gel}_{10s}=0.5\Phi_{3}$$

(5) $${Gel}_{10\min}=0.5\Phi_{3}$$

where, Φ_600_, Φ_300_, and Φ_3_ are the dial readings at 600, 300, and 3 rpm, respectively; Gel_10s_ is the maximum reading value at 3 rpm after standing for 10 s; Gel_10min_ is the maximum reading value at 3 rpm after standing for 10 min.

#### 4.3.3. Electrical Stability (ES) and High-Temperature, High-Pressure (HTHP) Filtration Tests

Different concentrations of OCD were added to the OBDFs, and the mixtures were stirred at 10,000 rpm for 20 min. The ES of the OBDF was measured using an electrical stability tester (DWY-2, Hengtaida Mechanical and Electrical Equipment Co., Ltd., Qingdao, China) at 50 °C. The electrode probe was placed in the middle of the OBDF, and the emulsion-breaking voltage was accorded. To ensure the accuracy of the data, the ES was measured three times, and the average value was calculated. Similarly, different concentrations of OCD were added to the OBDFs and stirred at 10,000 rpm for 20 min. The OBDFs were hot-rolled at 120 °C, 150 °C, and 180 °C for 16 h. After aging, the HTHP filtration loss was determined using an HTHP filtration tester (GGS71-B, Hengtaida Mechanical and Electrical Equipment Co., Ltd., Qingdao, China) at the respective aging temperatures and under a pressure of 3.5 MPa.

#### 4.3.4. Solid-Phase Settling Stability Test

White oil, commonly used as the base oil in OBDFs [30], was used to disperse solid particles for testing. Three types of white oil suspensions with different solid phases were prepared, each containing 15 wt% of Rev dust, 15 wt% of bentonite, and 15 wt% of barite. Different concentrations of OCD were added to each suspension and thoroughly mixed. For each suspension, 50 mL was taken, poured into a stoppered measuring cylinder, and shaken well. After allowing the samples to stand for 30 min, the effect of OCD on the suspension of solid phases in the white oil solution was observed.

### 4.4. Viscosity Reduction Mechanism Analysis

#### 4.4.1. Solid-Phase Wettability Test

Rev dust, bentonite, and barite were used to simulate the solid phases in high-density oil-based drilling fluids (OBDFs). Due to the strong hydrophilicity of these solid phases, they disperse well in ethanol, and since OCD is also easily soluble in ethanol, ethanol was chosen as the organic solvent to ensure effective interaction between the solid phases and OCD. First, ethanol suspensions were prepared for each of the three different solid phases, with each suspension containing 15 wt% Rev dust, 15 wt% bentonite, and 15 wt% barite. Then, a portion of each suspension was treated with 2 wt% OCD and stirred for 1 h. The suspensions were centrifuged at 2000 r·min^−1^ for 10 min, and the solid phases at the bottom were collected. The solid phases were dried at 85 °C, and after the liquid phase had completely evaporated, the dried solid phases were collected. The dried solid phases were compressed into tablets under 12 MPa pressure for 30 min. The contact angle of water droplets on the surface of the solid phases was measured using a contact angle goniometer (OCA25, Dataphysics, Filderstadt, Germany) to evaluate the effect of OCD on the wettability of the solid phases. The dosing rate was set to 0.5 µL/s, and the dosing volume was 3 µL. After the water droplet stabilized, the contact angle was photographed and measured. Each contact angle test for the solid phases with water was repeated three times, and the average value was taken to ensure the error was within ±0.5°.

#### 4.4.2. Solid-Phase Particle Size and Micromorphology Test

White oil suspensions were prepared with 15 wt% Rev dust, 15 wt% bentonite, and 15 wt% barite. The particle size changes of each suspension were monitored in real time using a focused beam reflectance measurement instrument (Particle Track G600, Mettler Toledo, Zurich, Switzerland). During the testing, 0.5 wt% OCD was added to the suspensions every 3 min. At the same time, an electron optical microscope (DM4 M, Leica, Wetzlar, Germang) was used to observe the effect of OCD on the solid-phase suspensions.

## Figures and Tables

**Table 1:** Preparation process and formula of the OBDF.

Order	Additives	Addition	Stirring Speed (rpm)	Stirring Time (min)	Function
1	White oil	240 mL	/	/	Base oil as continuous phase
2	Primary emulsifier	7.5 g	10,000	10	Helps in forming a stable oil–water emulsion
3	Secondary emulsifier	7.5 g	10,000	10	Helps in forming a stable oil–water emulsion
4	30% CaCl_2_	60 mL	10,000	20	Controls water activity and enhances water-in-oil emulsion formation
5	CaO	15 g	10,000	20	Adjusts the pH
6	Organoclay	6 g	10,000	20	Maintains the suspension of solids
7	Asphalt	7.5 g	10,000	20	Controls Fluid loss
8	Lignite	7.5 g	10,000	20	Controls Fluid loss
9	Bentonite	7.5 g	10,000	20	Deleterious solid phases
10	Rev dust	7.5 g	10,000	20	Deleterious solid phases
11	Barite	660 g	10,000	20	Increases the density of drilling fluid

## Data Availability

Data underlying the results presented in this paper are not publicly available at this time but may be obtained from the corresponding authors upon reasonable request.
